# Treatment of Prolapsing Hemorrhoids in HIV-Infected Patients with Tissue-Selecting Technique

**DOI:** 10.1155/2017/1970985

**Published:** 2017-03-05

**Authors:** Zhe Fan, Yingyi Zhang

**Affiliations:** Department of General Surgery, Dalian Third People's Hospital Affiliated to Dalian Medical University, Dalian, China

## Abstract

The aim of this retrospective study was to evaluate the outcome of a tissue-selecting therapy stapler (TST) for prolapsing hemorrhoids in HIV-infected patients. Sixty-two patients with stage III-IV hemorrhoidal prolapse were treated with TST by a single surgeon between June and November 2014. The TST group comprised 32 patients (4 females), and the TST + HIV group comprised 30 HIV-infected patients (3 females). Age, gender, and preoperative examination as well as intraoperative and postoperative features were assessed. There was no marked difference in hemorrhoidal prolapse between the TST and HIV + TST groups, except for patient satisfaction at 12 months. TST is an effective and safe technique for treatment of prolapsing hemorrhoids in HIV-infected patients.

## 1. Introduction

Hemorrhoids are a commonly encountered benign disease in anorectal surgery and 10%–20% of patients require surgery, especially those with severe prolapsing hemorrhoids [1, 2]. There are two methods to treat hemorrhoids: Milligan-Morgan hemorrhoidectomy (MMH) and the procedure for prolapse and hemorrhoids (PPH) [1], which have their advantages and disadvantages. MMH is used widely because it is an effective and definitive treatment; however, postoperative pain is the main disadvantage [3]. PPH has the advantage of reducing postoperative pain, hospitalization, and operating time [4], while bleeding and strictures are the main disadvantages [5]. Recently, the technique of a tissue-selecting therapy stapler (TST)—a segmental stapled hemorrhoidopexy [6]—emerged, which can reduce the complications of PPH.

An estimated 35 million people worldwide are affected by HIV infection, and 2.3 million new infections occur annually [7]. Perianal diseases usually require surgery in HIV-infected patients, who comprise 5.9–34% of HIV-infected patients [8].

The present study was conducted to evaluate whether TST was effective in patients with HIV infection.

## 2. Patients and Methods

### 2.1. Patients

We enrolled 62 patients with stage III-IV hemorrhoidal prolapse at the Third People's Hospital of Dalian, China, from June to November 2014. The TST group comprised 32 patients (28 males, 4 females; mean age 41 years, age range 21–59 years), and the TST + HIV group comprised 30 HIV-infected patients (27 males, 3 females; mean age 37 years, age range 23–59 years). The HIV-infected patients were selected following the standard CD4 > 350  cells/*μ*l and no medication treatment. The following data were collected: age, sex, hemorrhoidal stage, postoperative stenosis, intraoperative bleeding, postoperative bleeding, postoperative urine retention, and anal incontinence. Hemorrhoids were classified into the following stages [9]: (I) anal cushions protruding into the anal canal but maintaining at the proper level, (II) prolapse with bowel movements but with spontaneous reduction, (III) prolapse with bowel movements but requiring manual reduction, and (IV) prolapse but not reducible. Patients with chronic fissure, acute thrombosis, fixed fibrous external hemorrhoids, abscess, benign rectal disease, colorectal carcinoma, anal strictures, and severe primary diseases were not included in the study [10]. HIV-infected patients were selected on the basis of hemorrhoids being their only perianal disease. All patients underwent enteroscopy and treatment with the TST (Touchstone International Medical Science, Suzhou, China). Operations were set in the special infection operating room (OR), and surgeons wore single-use operating coats, single-use protective eyewear, and two pairs of gloves. During the operation time, anything in the OR could not be moved out and medical waste was marked HIV (+) postoperatively.

The postoperative HIV-infected patients were administrated by disease control and prevention (CDC); CD4 was tested once a month. If one's CD4 was lower than 350 cells/*μ*l, he would advise medication treatment including tenofovir disoproxil fumarate 300 mg/day, lamivudine 300 mg/day, and efavirenz 600 mg/day. The study was approved by the Institutional Review Board of Dalian Medical University. All of the procedures were conducted according to the guidelines of the Institutional Patients Care and Use Committee of Dalian Medical University and were approved by the Institutional Ethics Committee of Dalian Medical University. Informed consents were obtained from all patients.

### 2.2. TST

A cathartic agent of polyethylene glycol was administered in the evening before the operation, and prophylactic single-dose injections of metronidazole 0.5 g intravenously were administered at the time of anesthesia induction. Routine examinations such as laboratory testing, liver and kidney function tests, and other biochemical measures were carried out, and no abnormalities were found.

**Figure 1:**
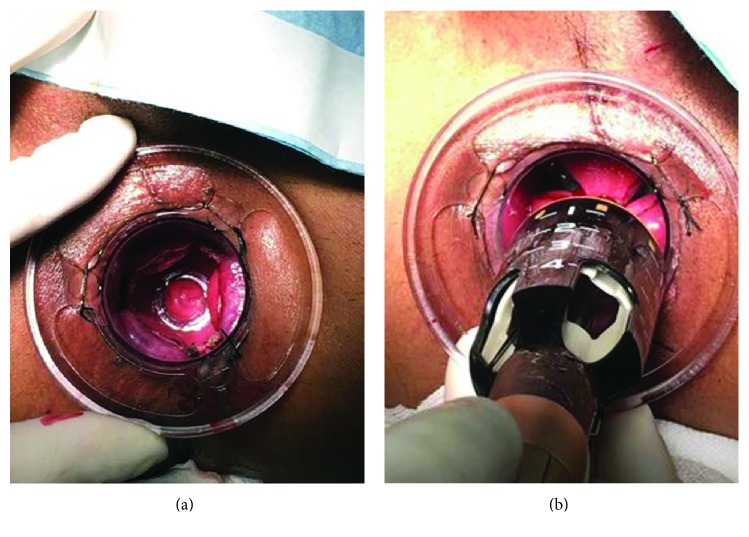
(a) The three-window anoscope without the obturator was inserted into the anus. (b) The 2/0 Vicryl suture was tied to the rod of the TST.

All operations were performed under epidural anesthesia, and all patients were in the lithotomy position. The surgical procedure was based on the technique of Lin et al. [6] and was performed by a single experienced surgeon as follows: TST consists of two rows of 33 mm diameter titanium staples; the obturator was inserted into the anus for full dilatation; the three-window anoscope with the obturator was inserted into the anus again; the obturator was pulled out (Figure 1(a)); the mucosa was sutured using a 2/0 Vicryl suture (Ethicon, Cincinnati, OH, USA), at 3-4 cm above the dentate line; the TST was opened to the maximum; the 2/0 Vicryl suture was tied to the rod of the TST (Figure 1(b)); the stapler was strained and fired; and the TST was removed from the anal canal.

Details of operating time, length of hospital stay, patient satisfaction, and perioperative complications (occurring up to postoperative day 30) were collected, as described previously [11].

### 2.3. Statistical Analysis

All data were analyzed by SPSS version 20.0 (SPSS, Chicago, IL, USA). Quantitative parameters were expressed as mean ± standard deviation. Student's *t*-test was used to compare the TST and TST + HIV groups, and a *χ*^2^ test was used to compare the numerical data between the two groups. *P* < 0.05 was considered significant.

## 3. Results

Patient characteristics are shown in Table 1. There was no significant difference in age, sex, and hemorrhoid grading between the TST and TST + HIV groups. There was no obvious difference in hemorrhoidal prolapse (according to Goligher's classification) between the groups.

Details of the intraoperative and postoperative periods are shown in Table 2. Intraoperative bleeding was 4.81 ± 1.55 ml in the TST group and 4.57 ± 1.33 ml in the TST + HIV group (*P* = 0.507). Operating time was 29.6 ± 6.5 min in the TST group and 30.4 ± 10.1 min in the TST + HIV group (*P* = 0.710). Postoperative bleeding was seen in 1/32 patients in the TST group and 1/30 patients in the TST + HIV group (*P* = 0.964). Postoperative urine retention was seen in 5/32 patients in the TST group and 3/30 patients in the TST + HIV group (*P* = 0.761). Length of hospital stay was 6.25 ± 2.08 days (ranged from 4 to 11 days) in the TST group and 6.30 ± 2.04 days (ranged from 4 to 11 days) in the TST + HIV group (*P* = 0.924). Patient satisfaction index at 6 months was 7.72 ± 1.14 in the TST group and 8.40 ± 1.07 in the TST + HIV group (*P* = 0.019). Prolapse recurrence at 12 months was seen in 1/32 patients in the TST group and 1/30 patients in the TST + HIV group (*P* = 0.964).

## 4. Discussion

Hemorrhoidal prolapse is a common disease in anorectal departments. Khubchandani [12] developed a modified circular stapled hemorrhoidopexy technique in 2012, which was named partial stapled hemorrhoidopexy. In China, the latter technique is called TST. In 2013, Lin et al. [6] gave a detailed description of the use of TST and also concluded that TST is a safe and effective procedure for grade III-IV hemorrhoids [13]. TST was invented based on the anal cushions and prolapse of the anal mucosa [13, 14]. Although the circular stapled hemorrhoidopexy was also based on the above theory, because of complications such as urgency [15] and anal stenosis [16], surgeons are often reluctant to use PPH. Recent studies have proven the merits of TST, treating prolapsing hemorrhoids without any significant risk [1, 3, 6, 10]. Therefore, TST can be used widely.

The rate of HIV infection is increasing gradually worldwide [17]. Nearly 19.7% of HIV-infected patients also have perianal diseases including hemorrhoids [18]. Therefore, it is necessary to perform a safe and effective operation with rapid recovery in these patients. Several studies have concluded that there is poor healing of anorectal wounds in patients with HIV infection [9, 19]. However, Dua et al. revealed a low complication rate for HIV-infected patients [20]. Therefore, we focused on whether TST is an effective technique for HIV-infected patients. A MEDLINE search for articles in the English language from 1975 to 2015 with the terms “tissue selecting technique” and “human immunodeficiency virus (HIV)/acquired immune deficiency syndrome (AIDS)” revealed no entries.

Taremwa et al. demonstrated that there was high prevalence of thrombocytopenia in HIV-infected patients, which increased bleeding [21]. We excluded the patients with abnormal routine blood examination results, and patients enrolled in the HIV + TST group had normal results, including normal thrombocyte counts. In our study, there was no obvious difference in intraoperative bleeding between the two groups. Previous studies have shown that TST is a safe technique [1, 3], and our research concluded that HIV-infected patients have a similar process in treatment, so TST is safe in HIV-infected patients.

Wang et al. reported intraoperative blood loss of 2.45 ± 0.57 ml [1], and Lin et al. reported a mean loss of 10 ml [3]. We conclude that this difference was related to different surgeons with different operation proficiencies. In our study, intraoperative bleeding (4.57 ± 1.33 ml) in the HIV + TST group was less than that in the TST group (4.81 ± 1.55 ml). This could be explained by a more careful operation being performed in the HIV-infected patients.

There were no significant differences between the TST and HIV + TST groups with respect to operating time, although it was longer in the latter. The longer time represents a more careful and cautious procedure. Only one previous study recorded the operating time of TST. The operating time for TST in our study was longer than that of Wang et al. (29.6 ± 6.5 versus 18.3 ± 5.6 min) [1]. There are two possible explanations for this difference: (1) the surgeons were at different points on the learning curve and (2) there were different recording procedures; for example, we usually washed the lower rectum, which was included in the operating time. Lin et al. reported a mean operating time of 25 min [3], which is close to our result. There was no significant difference in postoperative urine retention between the two groups: 5/32 (15.6%) patients in the TST group and 3/30 (10.0%) patients in the TST + HIV group, which were higher than 22/240 (9.17%) patients reported in another study [1]. We speculate that operating time and proficiency played important roles. Lim et al. [22] considered that anesthesia was a confounding factor.

In the present study, postoperative bleeding occurred in 1/32 (3.13%) and 1/30 (3.33%) patients in the TST and TST + HIV groups, respectively. Postoperative bleeding needs to be treated in the operating room. No studies have discussed this complication so far.

The mean hospital stay (6.25 ± 2.08 days) in our study was longer than that in the study of Wang et al. (5.3 ± 0.6 days). Lin et al. reported a mean hospital stay of 1.8 days using TST Starr plus [6]. In our hospital, checking out of the hospital often is after excretion, which may be a reason. Lim et al. [22] revealed that urinary retention was an important reason for hospital stay, and Law et al. [23] have suggested the role of postoperative pain.

There was no significant difference in recurrence of hemorrhoids between the TST and HIV + TST groups. Our 1-year recurrence rate (1/32 and 1/30 patients) was similar to that of Lin et al. (4/118 patients) [3]. Braini et al. found a 1-year recurrence rate of 7/189 patients after PPH [4]. TST has a low recurrence rate in patients with and without HIV infection.

Compared with the TST group, there was a high satisfaction index at 12 months in the HIV + TST group; we found that HIV-infected patients often had poor self-image and were too ashamed to go to a hospital. Prolapsing hemorrhoids are a particular concern to HIV-infected patients, so improving their symptoms leads to greater patient satisfaction. Because the wound is above the dentate line, there is no obvious postoperative pain [1, 13, 22], so we did not collect the data about postoperative pain.

## 5. Conclusion

We conclude that TST is a safe technique with a low complication rate and minor technical difficulties, especially for HIV-infected patients. There is a high satisfaction index in HIV-infected patients.

## Figures and Tables

**Table 1:** Demographic data for patients in the TST and TST + HIV groups.

		TST	TST + HIV	*P* value
Sex	Male/female	28/4	27/3	0.761
Age (yr)		41 ± 12	37 ± 11	0.176
Goligher's grade	III/IV	17/15	15/15	0.809

**Table 2:** Intraoperative and postoperative periods for patients in the TST and TST + HIV groups.

	TST	TST + HIV	*P* value
Intraoperative bleeding (ml)	4.81 ± 1.55	4.57 ± 1.33	0.507
Operating time (min)	29.6 ± 6.5	30.4 ± 10.1	0.710
Postoperative bleeding (patients/whole group)	1/32	1/30	0.964
Postoperative urine retention (patients/whole group)	5/32	3/30	0.761
Length of hospital stay (d)	6.25 ± 2.08	6.30 ± 2.04	0.924
Patient satisfaction index at 12 months	7.72 ± 1.14	8.40 ± 1.07	0.019
Prolapse recurrence at 12 months	1/32	1/30	0.964
